# Hydrogen-rich water improves sleep consolidation and enhances forebrain neuronal activation in mice

**DOI:** 10.1093/sleepadvances/zpad057

**Published:** 2023-12-30

**Authors:** Scott M Vincent, Melika Madani, Dante Dikeman, Kyle Golden, Naomi Crocker, Cameron Jackson, Sam P Wimmer, Mary Dover, Alexis Tucker, Cristina A Ghiani, Christopher S Colwell, Tyler W LeBaron, Alex Tarnava, Ketema N Paul

**Affiliations:** Department of Integrative Biology and Physiology, University of California Los Angeles, Los Angeles, CA, USA; Department of Integrative Biology and Physiology, University of California Los Angeles, Los Angeles, CA, USA; Department of Integrative Biology and Physiology, University of California Los Angeles, Los Angeles, CA, USA; Department of Integrative Biology and Physiology, University of California Los Angeles, Los Angeles, CA, USA; Department of Integrative Biology and Physiology, University of California Los Angeles, Los Angeles, CA, USA; Department of Integrative Biology and Physiology, University of California Los Angeles, Los Angeles, CA, USA; Department of Integrative Biology and Physiology, University of California Los Angeles, Los Angeles, CA, USA; Department of Integrative Biology and Physiology, University of California Los Angeles, Los Angeles, CA, USA; Department of Integrative Biology and Physiology, University of California Los Angeles, Los Angeles, CA, USA; Department of Pathology and Laboratory Medicine, David Geffen School of Medicine, University of California Los Angeles, Los Angeles, CA, USA; Department of Psychiatry and Biobehavioral Sciences, David Geffen School of Medicine, University of California, Los Angeles, Los Angeles, CA, USA; Department of Psychiatry and Biobehavioral Sciences, David Geffen School of Medicine, University of California, Los Angeles, Los Angeles, CA, USA; Department of Kinesiology and Outdoor Recreation, Southern Utah University, Cedar City, UT, USA; Molecular Hydrogen Institute, Enoch, UT, USA; Natural Wellness Now Health Products Inc, Maple ridge, BC, Canada; Department of Integrative Biology and Physiology, University of California Los Angeles, Los Angeles, CA, USA

**Keywords:** hydrogen-rich water, HRW, sleep, insomnia, latency, fragmentation, deprivation, cFos, hypnotic, septual nuclei

## Abstract

**Study Objectives:**

Sleep loss contributes to various health issues and impairs neurological function. Molecular hydrogen has recently gained popularity as a nontoxic ergogenic and health promoter. The effect of molecular hydrogen on sleep and sleep-related neural systems remains unexplored. This study investigates the impact of hydrogen-rich water (HRW) on sleep behavior and neuronal activation in sleep-deprived mice.

**Methods:**

Adult C57BL/6J mice were implanted with electroencephalography (EEG) and electromyography (EMG) recording electrodes and given HRW (0.7–1.4 mM) or regular water for 7 days ad libitum. Sleep–wake cycles were recorded under baseline conditions and after acute sleep loss. Neuronal activation in sleep- and wake-related regions was assessed using cFos immunostaining.

**Results:**

HRW increased sleep consolidation in undisturbed mice and increased non-rapid-eye movement and rapid-eye-movement sleep amount in sleep-deprived mice. HRW also decreased the average amount of time for mice to fall asleep after light onset. Neuronal activation in the lateral septum, medial septum, ventrolateral preoptic area, and median preoptic area was significantly altered in all mice treated with HRW.

**Conclusions:**

HRW improves sleep consolidation and increases neuronal activation in sleep-related brain regions. It may serve as a simple, effective treatment to improve recovery after sleep loss.

**Statement of Significance**

Over 10% of the global population struggles with sleep loss, and few effective treatments do not have deleterious side effects. This study presents hydrogen-rich water as a safe, accessible potential therapeutic avenue for treating sleep-related disorders.

## Introduction

Poor sleep is a hallmark of modern society. Nearly 30% of American adults average ≤6 hours of daily sleep [1], and more than 10% of the global population has experienced some form of insomnia [2]. This widespread problem has severe and complex consequences for individual health. Acute sleep loss generates proinflammatory responses [3], increases physiological stress [4], impairs memory [5], decreases insulin sensitivity [6], and may accelerate the progression of chronic complex diseases [7]. Chronic sleep loss is associated with significantly increased mortality [8]. There are global and local consequences of sleep loss in the brain and body [9, 10], and disrupted sleep is a risk factor and consequence of many disorders [11, 12]. In instances of comorbid mental health disorders and insomnia, improving sleep is often sufficient to improve symptoms of the comorbid disorder [13]. Behavioral interventions for improving sleep can be effective but are often inadequate to resolve common sleep disturbances. If behavioral interventions fall short, pharmaceutical hypnotics are often prescribed because they are fast-acting. However, undesirable side effects often accompany these drugs, and long-term use can lead to drug dependence [13]. Other drugs commonly prescribed to resolve poor sleep are re-purposed “off-label” drugs that have a range of biological impacts beyond sleep. The value of an intervention that improves sleep quality or reduces the consequences of sleep loss without deleterious side effects cannot be easily overstated.

Over the last decade, molecular hydrogen (H_2_ gas) has gained attention as a promising therapeutic with a wide range of potential benefits, such as the regulation of proinflammatory mediators [14] and the modulation of insulin sensitivity [15]. H_2_ has no reported cytotoxicity even at high concentrations and is thus widely accepted to have no deleterious side effects [16]. H_2_ can be administered via inhalation or dissolved in either saline or in water (hydrogen-rich water, HRW). Depending on the dose/concentration, oral ingestion of HRW may increase levels of H_2_ in the brain [17]. In rats, HRW dose-dependently increases H_2_ concentration in the blood [18]. Importantly, H_2_ appears to have region-specific influence on brain metabolism in humans [19]. Oral administration of HRW was more effective than inhalation of H_2_ at attenuating dopaminergic cell loss in a rat model of Parkinson’s Disease [20]. Recent work in humans demonstrates that HRW may increase alertness and cognitive function similarly to caffeine but likely through a different mechanism [19]. In the face of pharmacological or chemical challenges, HRW appears to act as a neuroprotectant in the hippocampus [21]. While numerous reports have demonstrated that HRW can favorably modulate various neurobiological processes and behavior, its effect of HRW on sleep remains unknown.

In this study, we tested the ability of 7 days of ad libitum access to HRW at 0.7–1.4 mM to alter baseline sleep–wake architecture and the response to acute sleep deprivation in wild-type C57BL/6J mice. We use polysomnography to assess several electrophysiological and behavioral markers of circadian activity and sleep pressure in freely moving mice. This randomized, within-participants investigation of HRW’s effect on sleep and wake behavior is the first of its kind. Separately, we performed a between-participants assessment of neuronal activation in known sleep- and wake-related brain regions following the HRW treatment regimen described above.

## Materials and Methods

### Animals

Adult C57BL/6J mice were maintained at the University of California Los Angeles under a 12–12 hours light–dark cycle (LD) in a light- and temperature-controlled study area overseen by the University Animal Research Committee and Division of Laboratory Animal Medicine. Food and water were provided ad libitum except when otherwise described. Sleep deprivation was performed by experts blinded to experimental conditions using gentle handling. Experiments were performed using the National Institutes of Health Guidelines for the Care and Use of Laboratory Animals and approved by the Institutional Animal Care and Use Committee. For a complete timeline of animal experiments see Supplementary Figures S1 and S2.

### Polysomnographic implantations

Adult C57BL/6J mice (postnatal week 10; *n* = 10; male = 6, female = 4) mice were implanted with EEG/EMG headmounts for polysomnographic (PSG) recording. As previously reported [22], mice were implanted with 4x EEG and 2x EMG electrodes under anesthesia. Two electrodes (frontal-parietal and ground) were located 1.5 mm anterior to bregma and 1.5 mm on either side of the central suture (EEG1). Two additional electrodes (parietal-occipital and common reference) were located 2.5 mm posterior to bregma and 1.5 mm on either side of the central suture (EE2). Electrical continuity between the screw electrode and headmount was achieved with silver epoxy. EMG activity was monitored using stainless-steel Teflon-coated wires inserted into the nuchal muscle. The headmount (2 × 3 pin grid array) was secured to the skull with dental acrylic. Seven days after surgery, mice were transferred to sound-attenuated chambers and connected to the data acquisition system. In the recording chambers, mice acclimated to a lightweight tether attached to a low-resistance commutator mounted above the cage for an additional 7 days before recording. Mice had free range of movement throughout all tethered experiments.

### HRW preparation

HRW was produced by adding magnesium-based tablets to 590 mL of deionized water in polycarbonate bottles. Elemental magnesium reacts with water to produce hydrogen by the following reaction: Mg + H_2_O → H_2_ + Mg(OH)_2_. Bottles were sealed and left overnight at 4°C for next-day administration. H_2_ levels of ~2 ppm (10 μL/g; 1.0 mM) and 2-hour half-life in glass administration bottles were confirmed by H_2_Blue titration assay (H_2_ Sciences; Henderson, NV). Tablets were provided by HRW Natural Health Products (New Westminster, BC, Canada).

### HRW administration

Mice were pseudo-randomly assigned to one of two groups to account for unintended order effects. Group 1 had ad libitum access to standard deionized water for 7 days, immediately followed by a 24-hour polysomnographic recording, and subsequent 6 hours of sleep deprivation by gentle handling and 18 hours of recovery sleep. Starting the following day, mice had ad libitum access to HRW for 7 days. The half-life of the gas was determined to be approximately two hours, and so the water was replaced every two hours during their active phase to maintain HRW concentrations of 1.0–2.0 ppm throughout the active phase of each day. Mice had access to the latest administered HRW throughout their inactive phase. Immediately following the 7th day of HRW treatment, another 24-hour recording was collected, followed by another 6 hours of sleep deprivation and 18 hours of recovery. Mice had ad libitum access to standard deionized water during the recording period and did not receive HRW. Group 2 underwent the opposite schedule to account for any potential order effects of the treatment condition. While receiving standard deionized water for 7 days, cages of both groups were gently disturbed every 2 hours during the active phase to control for any unintended effects of repeated bottle changes by the experimenter.

### Polysomnographic data acquisition and processing

Forty-eight-hour continuous PSG recordings consisting of 24-hour baseline, 6-hour sleep deprivation, and 18-hour recovery began at light onset, zeitgeber time (ZT) 0. Data acquisition were performed on a computer running polysomnographic software (Sirenia Acquisition, Pinnacle Technologies, Lawrence, KS). Signals were amplified and high-pass filtered (0.5 Hz) via a preamplifier. EEG signals were low-pass filtered with a 40 Hz cutoff and collected continuously at a sampling rate of 400 Hz. After collection, EEG and EMG waveforms were classified in 10-second epochs as: (1) wake (low-voltage, high-frequency EEG; high amplitude EMG); (2) non-rapid-eye movement (NREM) sleep (high-voltage, mixed-frequency EEG; low-amplitude EMG); or rapid-eye movement (REM) sleep (low-voltage EEG with a predominance of theta activity (6–10Hz); very low-amplitude EMG). All sleep scoring was performed by expert technicians blinded to the conditions of the experimental condition. EEG epochs determined to have artifacts (interference caused by scratching, movement, eating, or drinking) were excluded from analysis. Artifacts comprised less than five percent of all records used for analysis.

NREM relative delta power was calculated for each 2-hour epoch by dividing NREM-specific delta (0.5–4.0Hz) power by NREM-specific total power for each EEG separately. These calculations were completed for each condition and an example formula is provided below:


$$\begin{array}{l} Baseline\;NREM\;rDelta\;(ZT6\text{-}8)\;=\;\frac{EEG1\;delta\;power\;(ZT6\text{-}8)}{EEG1\;total\;power\;(ZT6\text{-}8)} \end{array}$$

Feature extraction was completed using a proprietary MATLAB script built in-house for the purpose of automatically extracting >40 sleep features of interest from data obtained using the Sirenia Acquisition platform described above. This script is available for free use upon request.

### Animal treatment and immunofluorescence

Adult C57BL/6J mice (postnatal week 12; *n* = 33; male = 16, female = 17) were pseudo-randomly assigned to one of the following groups: (1) undisturbed mice with ad libitum access to standard deionized water, CON; (2) sleep-deprived mice with ad libitum access to standard deionized water, CON + DEP; (3) undisturbed mice with ad libitum access to HRW, HRW; and (4) sleep-deprived mice with ad libitum access to HRW, HRW + DEP. HRW and HRW + DEP mice received 7 days of ad libitum access to HRW as described above. CON and CON + DEP mice had water bottles disturbed as described above. On day 7 of treatment, CON and HRW groups were allowed to sleep for 6 hours from ZT 0–6, while CON + DEP and HRW + DEP groups were sleep deprived for 6 hours by gentle handling. All groups were perfused at ZT6 (mean = ZT 6.61 ± 0.2). The difference in mean perfusion time between any two groups was no more than 7 minutes. Mice were euthanized with Euthasol (150 mg/kg), perfused with 10 mL of 1× PBS, and then 10 mL of 4% paraformaldehyde (PFA). Brains were dissected out and post-fixed with 4% PFA at 4°C overnight, then transferred into a solution of 15% sucrose in 1×PBS. Fixed brains are preserved in 15% sucrose in 1×PBS + 0.1% Na azide until ready to cut.

Floating coronal sections (50 μm) were obtained on a cryostat (Leica) and collected sequentially into 24-well plates containing 1:1 ice-cold glycerol:PBS and stored at −20°C. A single person collected all the sections and brains from all four experimental conditions were sectioned during any single cryostat session to ensure that tissue collection conditions did not disproportionately influence any group(s). Sections were paired along the anterior–posterior axis and stained. First, sections were blocked for 1 hour at room temperature (1% BSA, 0.3% Triton X-100, 10% normal donkey serum in 1×PBS) and then incubated overnight at 4°C with a rabbit polyclonal antiserum against cFos (1:1000, Cell Signaling) followed by a Cy3-conjugated donkey-anti-rabbit secondary antibody (1:300, Jackson ImmunoResearch Laboratories, Bar Harbor, ME). Sections were mounted and coverslips applied with Vectashield mounting medium containing DAPI (4ʹ -6-diamidino-2-phenylinodole; Vector Laboratories, Burlingame, CA), and visualized on a Zeiss AxioImager M2 microscope (Zeiss, Thornwood NY) equipped with an AxioCam MRm and the ApoTome imaging system. Images (2–4 per each animal and condition) for counting were acquired using a 20X objective and the Tile tool of the Zeiss Zen digital imaging software. Regions of interest (ROIs) were defined and manually traced in ImageJ (National Institutes of Health; LOCI, University of Wisconsin) using the Allen Institute’s Mouse Brain Atlas (University of Washington) for reference. cFos-positive cells in the defined ROIs were counted with the aid of the cell counter plugin of ImageJ in at least two consecutive sections by two experts masked to the conditions. The values obtained by each observer in the 2–4 sections were averaged to obtain one value for each animal.

### Statistical analysis

All data were analyzed using GraphPad Prism version 9.3.1. Sleep architecture data were analyzed using paired *t*-tests. Two-way repeated measures ANOVA was used to analyze the effects of time and treatment on NREM and REM sleep during baseline and recovery recordings. Slow wave activity (SWA) was assessed across time and treatment conditions and, because SWA was assessed during NREM sleep only and there were some 2-hour epochs where NREM sleep did not occur, data were analyzed using a mixed-effects model. Multiple comparisons were corrected for using Bonferroni’s method. For cFos immunofluorescence data, the total number cFos + cells were compared between groups using two-way ANOVA with sleep condition and treatment as factors. Post hoc comparisons between groups were made using Bonferroni’s multiple comparisons test or Benjamini and Hochberg’s method for controlling false discovery rate. Statistical significance was set at *p* < 0.05. Exact *p*-values and associated statistics are reported in figures and tables.

## Results

### HRW decreases time to sleep onset after light onset in undisturbed mice

**Figure 1.**
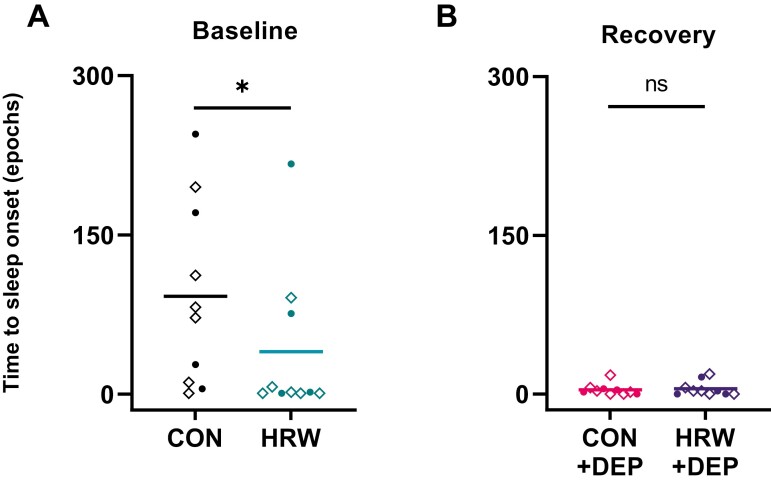
HRW treatment is associated with decreased time to sleep onset. (A) Paired *t*-test with Benjamini and Hochberg post-test revealed a significant effect (*p* = 0.0313, *t* = 2.549, *df* = 9) of HRW treatment on time to sleep onset in undisturbed mice. (B) Following sleep deprivation, no effect of HRW was observed (*p* = 0.5765, *t* = 0.5795, *df* = 9). Baseline time to sleep onset is the time it takes an animal to accumulate a bout of NREM sleep following the beginning of the inactive phase (lights on, ZT 0). Recovery sleep latency is the time it takes to accumulate a bout of NREM sleep following the end of sleep deprivation at ZT 6. A NREM bout is two or more adjacent 10-second NREM epochs. Horizontal bars represent mean. Solid circles are female. Diamonds are male.

Time to sleep onset after light onset is an indicator of sleep efficiency used across species [22–24]. During ad libitum sleep in a 12:12 LD cycle, sleep after light onset (often called “sleep latency” in mouse models) is a standard and commonly accepted measurement of sleep efficiency and is part of the diagnostic criteria for sleep disorders like insomnia [25]. Several factors, including age [26], presence or absence of chronic pain [27], alcohol use [28], and exercise [29] can influence time to sleep onset. Here, we tested the hypothesis that HRW treatment would be sufficient to promote sleep in mice. During baseline, time to sleep onset is defined as the amount of time it takes for an animal to accumulate at least one bout (20 seconds or more) of NREM sleep after lights turn on at ZT 0. Time to sleep onset during recovery is the amount of time it takes an animal to accumulate at least one bout of NREM sleep following the termination of sleep deprivation at ZT 6. Following 7 days of ad libitum access to HRW throughout their active phase, paired t-test revealed a statistically significant difference in time to sleep onset (*p* = 0.0313, *t* = 2.549, *df* = 9) during the undisturbed, baseline condition, with HRW-treatment reducing sleep onset by >50% (Figure 1A). Following 6 hours of acute sleep deprivation by gentle handling, we what we believe to be a ceiling effect in time to sleep onset as a marker for sleep efficiency (i.e. mice cannot fall asleep faster than “immediately”) and thus observe no effect of HRW during recovery (Figure 1B).

### HRW treatment does not alter normal sleep amount

**Figure 2.**
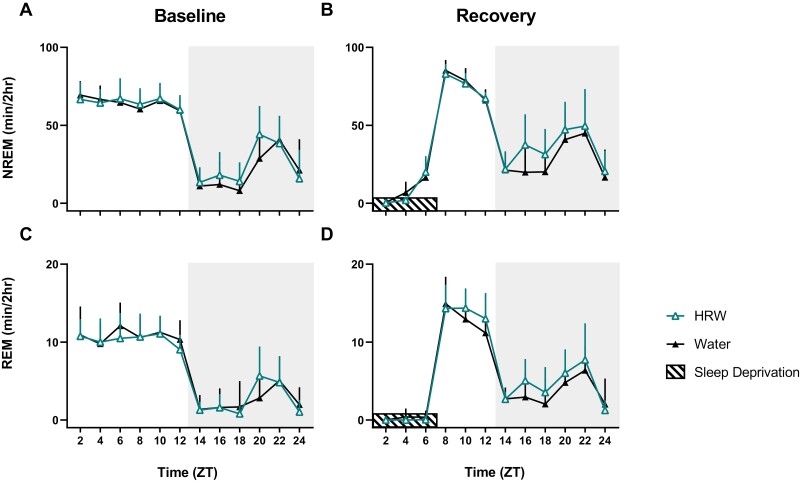
No effect of HRW on organization of sleep behavior. (A-D) Within-participants comparisons by two-way ANOVA with Geisser-Greenhouse correction reveal no significant effect of HRW on the distribution of NREM or REM sleep during 24-hour baseline or 18-hour recovery conditions. Multiple comparisons were corrected using Bonferroni’s multiple comparison test, with individual variance computed for each comparison. Icons represent the mean and error bars are standard deviation. Shaded boxes label the dark phase. See Table 1 for statistics.

Mice have polyphasic sleep—while they accumulate most of their sleep during the inactive (light) phase, they still achieve significant amounts of sleep during their active (dark) phase [30]. The sleep architecture of undisturbed C57BL/6J and their response to acute total sleep deprivation (increased NREM sleep, for instance) are well documented and highly stable [22, 31, 32]. During HRW treatment, two-way ANOVA reveals no effect of HRW on the robust typical distribution of NREM or REM throughout baseline recording, during sleep deprivation, or during the 18-hour recovery period (Figure 2A–D, Table 1).

**Table 1.** No Effect of HRW on Organization of Sleep Behavior

Source of variation	SS	F(DFn, DFd)	*P*-value	Summary
*Baseline NREM*				
Time	125 152	*F* (11, 198) = 73.25	*p *< 0.0001	****
Treatment	251.5	*F* (1, 18) = 0.9161	*p *= 0.3512	ns
Time × treatment	1652	*F* (11, 198) = 0.9666	*p *= 0.4782	ns
*Baseline REM*				
Time	4266	*F* (11, 198) = 70.45	*p *< 0.0001	****
Treatment	2.017	*F* (1, 18) = 0.09647	*p *= 0.7597	ns
Time × treatment	68.77	*F* (11, 198) = 1.136	*p *= 0.3353	ns
*Recovery NREM*				
Time	168 826	*F* (11, 198) = 96.70	*p *< 0.0001	****
Treatment	658.4	*F* (1, 18) = 2.826	*p *= 0.1100	ns
Time × treatment	2140	*F* (11, 198) = 1.225	*p *= 0.2720	ns
*Recovery REM*				
Time	6287	*F* (11, 198) = 86.55	*p *< 0.0001	****
Treatment	22.41	*F* (1, 18) = 2.970	*p *= 0.1019	ns
Time × treatment	60.53	*F* (11, 198) = 0.8333	*p = *0.6069	ns

Within-participants comparisons by repeated measures of two-way ANOVA revealed no significant effect of HRW on the distribution of NREM or REM sleep during 24-hour baseline or 18-hour recovery conditions. Multiple comparisons were corrected for using Bonferroni’s multiple comparison test, with individual variance computed for each comparison.

### HRW increases total NREM and REM sleep amount following sleep deprivation

**Figure 3.**
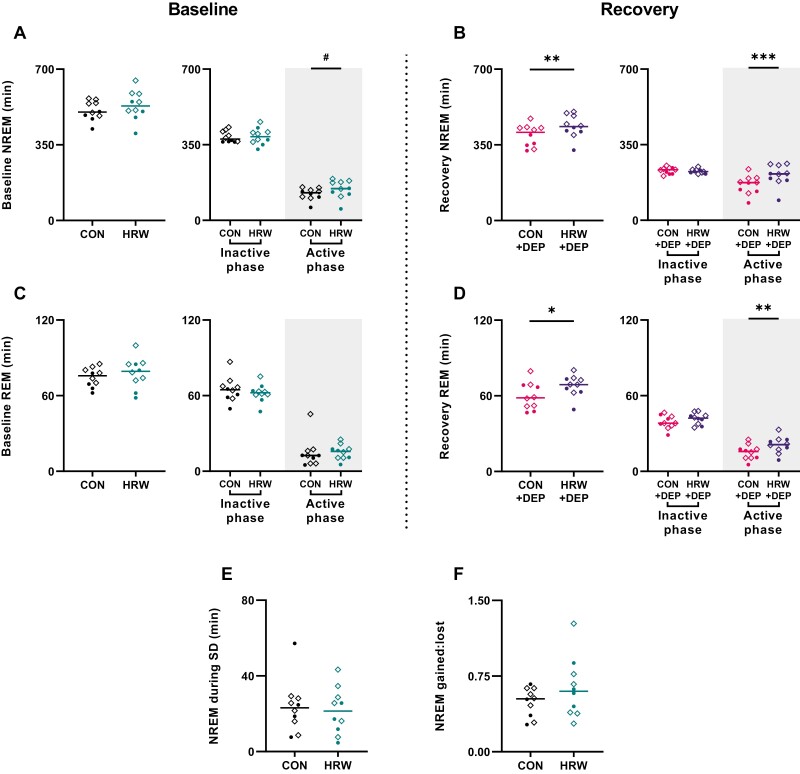
HRW significantly increases NREM and REM sleep following sleep deprivation. (A, C) Paired t-tests with Benjamini and Hochberg post-test suggest no effect of HRW on total NREM or REM amount in baseline recordings, though NREM active phase differences between treatment groups approach significance (#; *p* = 0.0524, *t* = 0.200, *df* = 9). (B, D) Paired t-test suggests that following 6 hours of sleep deprivation, HRW-treated mice accumulated more NREM (*p* = 0.0012, *t* = 4.626, *df* = 9) and REM sleep (*p* = 0.0145, *t* = 3.021, *df* = 9) - this effect is specific to the active phase for both NREM (*p* = 0.0003, *t* = 5.767, *df* = 9) and REM sleep (*p* = 0.0045, *t* = 3.757, *df* = 9) during the recovery from sleep deprivation. (E) Paired t-test revealed no differences in the effectiveness of sleep deprivation between HRW-treated and control conditions (*p* = 0.725, *t* = 0.3804, *df* = 9). (F) Despite differences in recovery sleep and approximately equivalent sleep deprivations, we observe no significant differences in NREM gained-to-lost ratio (*p* = 0.2078, *t* = 1.357, *df* = 9). Horizontal bars represent mean. Circles are female. Diamonds are male. Shaded box labels dark phase.

In adult humans, total sleep has a significant impact on all-cause mortality [8] and is an important clinical endpoint for sleep disorders [25] and several complex chronic conditions [33, 34]. Recommendations from the American Academy of Sleep Medicine reflect decades of sleep research and state that fewer than 7 hours of sleep per night is inadequate to sustain health. In models where electrophysiology is not available, behavioral endpoints (quiescence, recumbent posture, increased arousal threshold, etc.) frequently rely on total sleep amount as a primary endpoint to assess sleep quality. In our polysomnographic investigation of mouse sleep, we observe no significant effect of HRW on NREM or REM sleep amount during baseline conditions (Figure 3, A and C); however, a slight increase in NREM sleep during the baseline active phase is near significance (*p* = 0.0524, *t* = 2.233, *df* = 9). We find that HRW treatment was associated with an increase in NREM (*p* = 0.0003, *t* = 5.767, *df* = 9) and REM (*p* = 0.0045, *t* = 3.757, *df* = 9) sleep amount (Figure 3, B and D), despite equivalent sleep deprivation interventions (Figure 3E). While there are no significant differences in the often-used NREM gained-to-lost ratio (Figure 3F), this may be due to the endpoint’s insensitivity to phase-specific effects (Figure 3, B and D).

### HRW increases sleep consolidation in undisturbed mice

**Figure 4.**
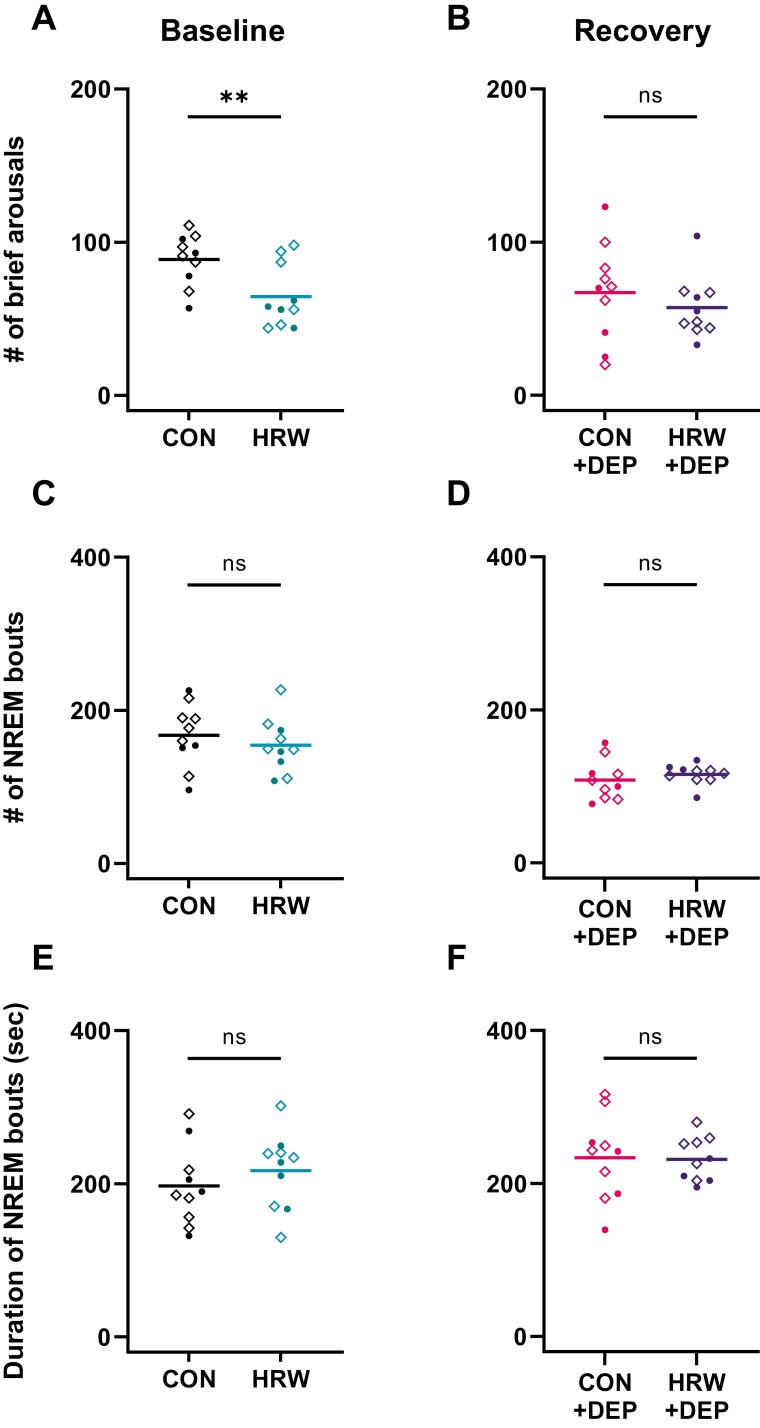
HRW treatment is associated with a significant reduction in brief arousals. (A-B) Paired *t*-test with Benjamini and Hochberg post-test reveals a significant effect of HRW treatment on brief arousals during 24-hour baseline recording (*p* = 0.0045, *t* = 3.758, *df* = 9) but not in the 18-hour recovery period following sleep deprivation (*p* = 0.2477, *t* = 1.236, *df* = 9). (C-F) Despite differences in brief arousals, paired *t*-test reveals no significant differences in the number of NREM bouts each animal experiences during baseline (*p* = 0.4535, *t* = 0.7835, *df* = 9) nor recovery (*p* = 0.3838, *t* = 0.9154, *df* = 9) nor the duration of those bouts during baseline (*p* = 0.4379, *t* = 0.8118, *df* = 9) or recovery (*p* = 0.9175, *t* = 0.1066, *df* = 9). A brief arousal is counted each time a single 10-second epoch of wake interrupts a bout of NREM sleep. A bout is two or more adjacent 10-second epochs of the same arousal state (NREM, REM, and wake). Horizontal bars represent mean. Solid circles are female. Diamonds are male.

Frequent awakenings and chronically poor sleep consolidation are hallmarks of some neurodegenerative diseases [33, 34] and sleep apnea [35]. Previous reports demonstrate that significant sleep fragmentation challenges can impact process S and sleep-dependent physiological processes without significantly changing daily rhythms [36, 37]. To assess changes in fragmentation, we evaluated (1) the number of brief arousals (periods of wake lasting ≤ 10 seconds, interrupting NREM), (2) the number of NREM bouts, and (3) the duration of NREM bouts. Paired *t*-test with Benjami and Hochberg post-test reveals a significant effect of treatment condition on brief arousals in undisturbed mice (Figure 4A), with mice experiencing a ~30% reduction in brief arousals following 7 days of ad libitum HRW treatment.

We also report a reduction in the number of NREM bouts (Figure 4B), and a non-significant increase in the duration of NREM bouts (Figure 4C) of HRW-treated baseline mice. These results suggest that HRW treatment may be meaningfully associated with improved NREM sleep consolidation in undisturbed mice. There is no significant effect of HRW on measures of sleep fragmentation during recovery sleep. Importantly, we do observe a significant, expected effect of sleep deprivation on NREM bouts, with sleep-deprived mice experiencing fewer NREM bouts (*p* < 0.0001, *t* = 5.968, *df* = 19) of greater average length (*p* < 0.0443, *t* = 2.154, *df* = 19). This previously known effect of sleep deprivation serves as an important positive control.

### HRW treatment does not alter NREM delta features

**Figure 5.**
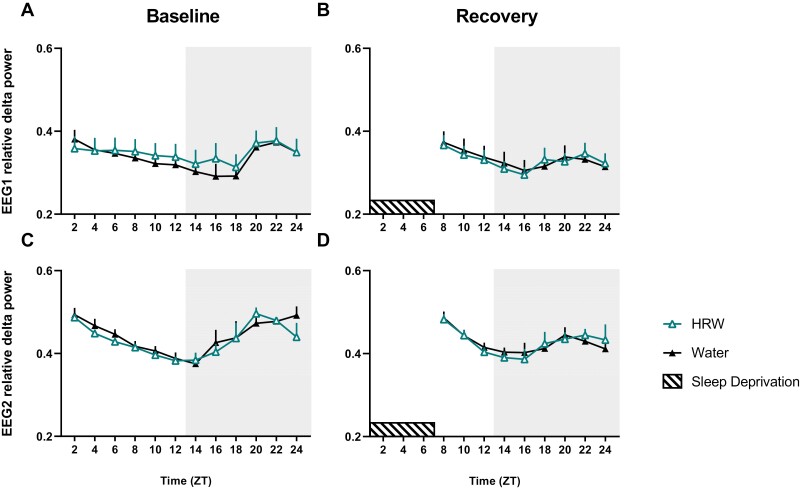
HRW does not alter relative delta power during baseline or recovery sleep. Mixed-effects analysis with Bonferroni post-test reveals no effect of HRW on relative delta power during baseline or during recovery from sleep deprivation. Twenty-four-hour distribution of SWA presented as relative delta power in HRW (outlined triangle) and water-treated (black triangle) mice. Icons represent the mean relative delta power for each animal during a 2-hour window, and error bars are standard error (SEM). Shaded box labels dark phase. Dashed bar represents the 6-hour sleep deprivation period.

Slow waves are low frequency (delta band, 0.5–4.0Hz), high amplitude oscillations that dominate the deepest stages of NREM sleep, whose changes in power are believed to reflect changes in sleep pressure. Measured in our study using EEG and presented as a fraction of total power, SWA is a commonly used metric for homeostatic dynamics in humans and animal models where EEG is currently available. Following sleep deprivation, mice demonstrate an expected rebound in NREM relative delta power (SWA) in EEG2 (Supplementary Figure S3), but mixed-effects analysis with Bonferroni post-test used to account for multiple reveals no significant differences across treatment conditions (Figure 5, Table 2).

**Table 2.** HRW Treatment Does Not Alter Slow Wave Activity During Baseline or Recovery Sleep

Fixed effects (type III)	F(DFn, DFd)	*P*-value	Summary
*EEG1—baseline*			
Time	***F* (2.591, 44.29) = 7.513**	*p* *** *= 0.0006**	**
Treatment	*F* (1, 19) = 0.2014	*p *= 0.6587	ns
Time × treatment	*F (11, 188) = 1.348*	*p *= 0.2008	ns
*EEG1—recovery*			
Time	***F* (3.179, 54.85) = 9.492**	*p* *** *< 0.0001**	****
Treatment	*F* (1, 18) = 0.002776	*p *= 0.9586	ns
Time × treatment	*F* (8, 138) = 0.8755	*p *= 0.5389	ns
*EEG2—baseline*			
Time	***F* (3.316, 53.96) = 16.91**	*p* *** *< 0.0001**	****
Treatment	*F* (1, 18) = 0.4276	*p *= 0.5214	ns
Time × treatment	*F* (11, 179) = 0.9781	*p *= 0.4680	ns
*EEG2—recovery*			
Time	***F* (1.291, 22.28) = 9.534**	*p* *** *= 0.0031**	**
Treatment	*F* (1, 18) = 0.0006621	*p *= 0.9798	ns
Time × treatment	*F* (8, 138) = 0.5450	*p *= 0.8208	ns

Mixed-effects analysis with multiple comparisons and Bonferroni’s post-test reveals an expected main effect of time during baseline and recovery sleep, but no effect of HRW. Significant results are in bold.

### The effect of HRW on cellular activity in known sleep and arousal nuclei

HRW may have region-specific influence on central processes [21, 38] in rodent models, and recent work in humans shows that high-dose HRW can increase choline‐to‐creatine ratio levels in certain brain regions and therefore alter brain metabolism [19]. It is unclear whether HRW influences the brain by meaningfully increasing H_2_ in the brain or through some second messenger [39].

In a separate study, we assessed changes in the expression of the immediate early gene cFos as a proxy for neuronal activity in several known sleep regulatory forebrain structures across four conditions: undisturbed, untreated mice (CON); sleep deprived, untreated mice (CON + DEP) undisturbed, HRW-treated mice (HRW); and sleep deprived, HRW-treated mice (HRW + DEP).

**Figure 6.**
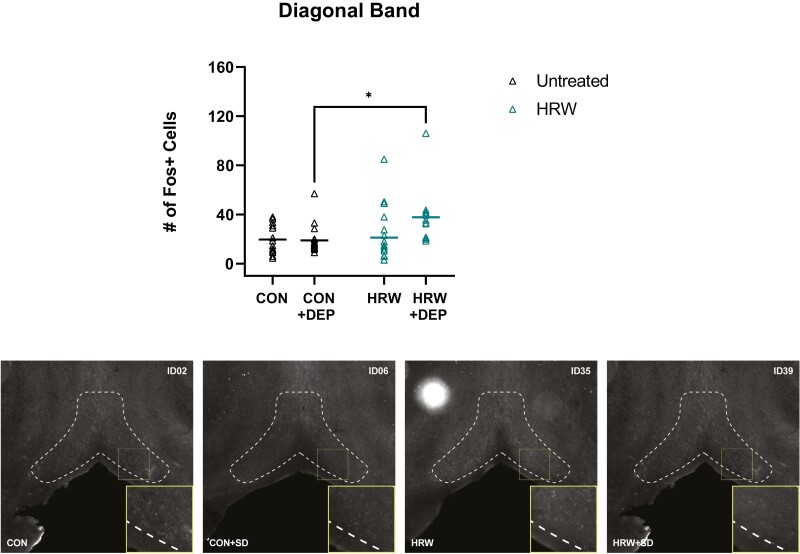
Moderate effect of HRW in the diagonal band is exclusive to recovery sleep. Total number of cFos + cells in the diagonal band. (top) A two-way analysis reveals a main effect of treatment (*p* = 0.0314, *F*(1,57) = 4.866). Bonferroni post-test reveals that the moderate effect of treatment in the DB is specific to recovery sleep (*p* = 0.0424, *t* = 2.794, *df* = 57). (Bottom) Representative coronal sections of diagonal band, boundaries outlined in dotted lines. Horizontal bars represent mean. See Table 3 for complete two-way ANOVA table. *, *p* < 0.05.

**Figure 7.**
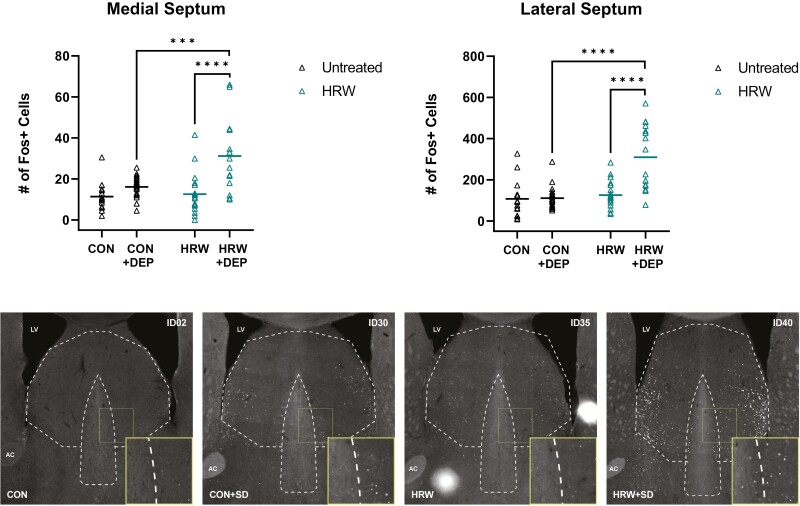
HRW-treated mice have significantly increased cFos + cells in septal nuclei. Total number of cFos + cells in the septal nuclei. (top, left; medial septum) A two-way analysis reveals a main effect of treatment (*p* = 0.0015, *F*(1,69) = 10.87) and sleep deprivation (*P* = 0.0015, *F*(1,69) = 22.18), and a statistically significant interaction of sleep and treatment conditions (*p* = 0.0067, *F*(1,69 = 7.825) in the MS. Bonferroni post-test reveals a significant effect of sleep deprivation in HRW-treated mice (*p* < 0.0001, *t* = 5.046, *df* = 69) and a significant effect of HRW in undisturbed mice (*p* = 0.0003, *t* = 4.373, *df* = 69). (top, right; lateral septum) A two-way analysis reveals a main effect of treatment (*p* < 0.0001, F(1,67) = 22.36) and sleep deprivation (*p* = 0.0001, *F*(1,67) = 16.55), and a statistically significant interaction of sleep and treatment conditions (*p* = 0.0002, *F*(1,67 = 15.35) in the LS. Bonferroni post-test reveals a significant effect of sleep deprivation in HRW-treated mice (*p* < 0.0001, *t* = 5.552, *df* = 67) and a significant effect of HRW in undisturbed mice (*p* < 0.0001, *t* = 6.224, *df* = 67). (Bottom) Representative coronal sections of septal nuclei, boundaries outlined in dotted lines. Horizontal bars represent mean. See Table 3 for complete two-way ANOVA table. ***, *p* < 0.001; ****, *p* < 0.0001.

**Figure 8.**
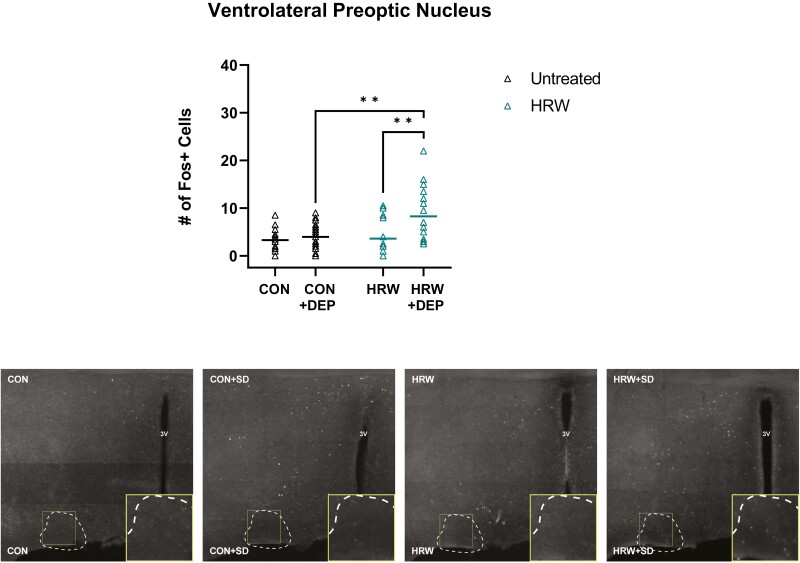
HRW-treated mice have significantly increased cFos expression in VLPO. Total number of cFos + cells in the ventrolateral preoptic nucleus. (Top) A two-way analysis reveals a main effect of treatment (*p = *0.0113, *F*(1,68) = 6.785) and sleep deprivation (*p* = 0.0037, *F*(1,68) = 9.051), and a statistically significant interaction of sleep and treatment conditions (*p* = 0.0277, *F*(1,68) = 5.059) in the VLPO. Bonferroni post-test reveals a significant effect of sleep deprivation in HRW-treated mice (*p* < 0.0001, *t* = 5.046, *df* = 69) and a significant effect of HRW in undisturbed mice (*p* = 0.0003, *t* = 4.373, *df* = 69). (bottom) Representative coronal sections of the VLPO, boundaries outlined in dotted lines. Horizontal bars represent mean. See Table 3 for complete two-way ANOVA table. *, *p* < 0.05; **, *p* < 0.01.

**Figure 9.**
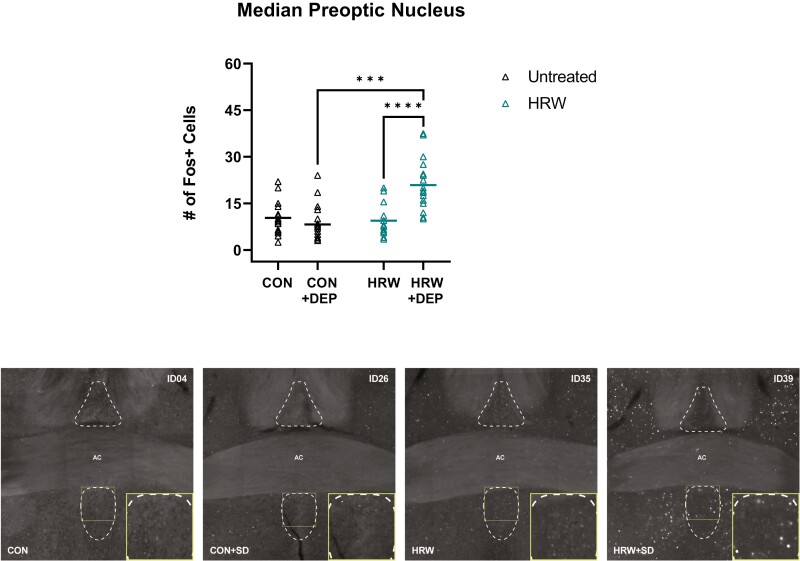
HRW-treated mice have significantly increased cFos induction in MnPO. Total number of cFos + cells in the median preoptic nucleus. (top) A two-way analysis reveals a main effect of treatment (*p = *0.0002, *F*(1,66) = 15.43) and sleep deprivation (*p* = 0.0027, *F*(1,66) = 9.697), and a statistically significant interaction of sleep and treatment conditions (*p* < 0.0001, *F*(1,66) = 20.52) in the MnPO. Bonferroni post-test reveals a significant effect of sleep deprivation in HRW-treated mice (*p* < 0.0001, *t* = 5.109, *df* = 66) and a significant effect of HRW in undisturbed mice (*p* < 0.0001, *t* = 6.475, *df* = 66). (bottom) Representative coronal sections of the MnPO, boundaries outlined in dotted lines. Horizontal bars represent mean. See Table 3 for complete two-way ANOVA table. ****, *p* < 0.0001.

We observe no effect of HRW on the number of cFos + cells in the forebrain diagonal band neurons (DB, Figure 6). While there was a significant effect of HRW treatment in the lateral septum (LS, Figure 7), medial septum (MS, Figure 7), the ventrolateral preoptic area (VLPO, Figure 8), and the median preoptic area (MnPO, Figure 9). We also observe a significant effect on cFos immunoreactivity of 6 hours of acute sleep deprivation on the MS, LS, VLPO, and MnPO. Results in the MS and LS are consistent with previous reports [40], but the increase in cFos immunopositive cells elicited by sleep deprivation in the VLPO and MnPO does not appear to have been previously reported in mice, despite numerous reports of sleep–active neurons in these areas. Finally, two-way analysis suggests a main effect of treatment in all ROIs, and a main effect of sleep in the MS, LS, VLPO, and MnPO (Table 3). Bonferroni post-test reveals multiple comparisons with statistically significant differences (Figures 7–9) and that a moderate effect of treatment in the DB is specific to recovery sleep (Figure 6).

**Table 3.** Two-way ANOVA Statistics for Brain Regions of Interest

Source of variation	SS	F(DFn, DFd)	*P* value	Summary
*Diagonal band (DB)*				
Sleep	933.4	*F* (1, 57) = 2.951	*p *= 0.0913	ns
Treatment	1539	*F* (1, 57) = 4.866	*p* = 0.0314	*
Sleep × treatment	1089	*F* (1, 57) = 3.444	*p* = 0.0687	ns
*Medial septum (MS)*				
Sleep	2372	*F (1, 69) = 22.18*	*p *< 0.0001	****
Treatment	1163	*F* (1, 69) = 10.87	*p* = 0.0015	**
Sleep × treatment	837	*F* (1, 69) = 7.825	*p *= 0.0067	**
*Lateral septum (LS)*				
Sleep	153 494	*F* (1, 67) = 16.55	*p *= 0.0001	***
Treatment	207 405	*F* (1, 67) = 22.36	*p *< 0.0001	****
Sleep × treatment	142381	*F* (1, 67) = 15.35	*p *= 0.0002	***
*Ventrolateral preoptic area (VLPO)*				
Sleep	127.3	*F* (1, 68) = 9.051	*p *= 0.0037	**
Treatment	95.42	*F* (1, 68) = 6.785	*p *= 0.0113	*
Sleep × treatment	71.14	*F* (1, 68) = 5.059	*p *= 0.0277	*
*Median preoptic area (MnPO)*				
Sleep	366.8	*F* (1, 66) = 9.697	*p *= 0.0027	**
Treatment	583.7	*F* (1, 66) = 15.43	*p *= 0.0002	***
Sleep × treatment	776.3	*F* (1, 66) = 20.52	*p *< 0.0001	****

A two-way analysis suggests a main effect of treatment in all regions of interest, and a main effect of sleep in the MS, LS, VLPO, and MnPO. Bonferroni post-test reveals multiple comparisons with statistically significant differences (see Figures 7–9) and that the moderate effect of treatment in the DB is specific to recovery sleep (Figure 6).

## Discussion:

We conducted a randomized, within-participants investigation of HRW and sleep–wake behavior in adult wild-type C57BL/6J mice to test the hypothesis that HRW may be sufficient to alter sleep–wake architecture. Following 7 days of ad libitum access to HRW during their active phase, 24-hour baseline polysomnography was collected, followed by 6 hours of sleep deprivation and a subsequent 18-hour recovery period. Mean latency to sleep onset was dramatically reduced by >50% following 7 days of HRW treatment (Figure 1A) without altering daily rhythms of sleep–wake behavior or the typical structure of recovery sleep (Figure 2). For context, some drugs currently prescribed for insomnia may only decrease sleep latency by less than 20% [41, 42]. We also observed a significant reduction of brief arousals in undisturbed mice during baseline (Figure 4A) but not after sleep deprivation (Figure 4B) where brief arousals were significantly reduced, as expected, in the control treatment condition. This reduction in brief arousals in HRW-treated mice was not accompanied by significant changes to the total number of NREM bouts or their duration (Figure 4, C–F). Total NREM and REM sleep amounts were unchanged during baseline of each treatment condition (Figure 4, A and C). Note that brief arousals were counted without ending the “interrupted” sleep bout.

Following sleep deprivation, mice in the HRW-treatment condition experienced significantly increased NREM (Figure 3B) and REM (Figure 3D) sleep which appears to have been principally driven by differences in active-phase sleep behavior. It is possible that the effect of HRW on recovery sleep is specific to the active phase because sleep pressure is already near its ceiling immediately following 6 hours of sleep deprivation. While direct observation of HRW half-life post-HRW-administration in mice (by gas chromatography, for example) has not been previously reported, data from other model species suggests that H_2_ persists in the body for a few hours, though its protective effects post-administration in small mammals may last several days [43]. While we report changes in behavioral markers of sleep pressure (time to sleep onset, NREM amount, and sleep consolidation), a mixed-effects analysis with multiple comparisons and Benjamini and Hochberg correction reveals no effect of HRW on NREM relative delta power during undisturbed conditions nor during the recovery from sleep loss (Figure 5). It may be the case that SWA is not a sensitive enough marker to reflect the subtle changes in sleep pressure induced by HRW. It may also be that direct activity changes in sleep regulatory systems unrelated to sleep homeostasis drive the effect of HRW on sleep.

To elucidate the effect of HRW on several sleep-related forebrain nuclei, we performed a between-participants mapping of cFos immunopositive cells in forebrain, hypothalamic, and midbrain structures across four groups: undisturbed, untreated mice (CON); sleep deprived, untreated mice (CON + DEP) undisturbed, HRW-treated mice (HRW); and sleep deprived, HRW-treated mice (HRW + DEP). We report here region-specific changes in sleep- and arousal-related forebrain structures. Three-way ANOVA with Bonferroni post-test reveals that changes in neuronal activation by HRW were largely unaffected by sex (Supplementary Table S1).

Septal nuclei are functionally and chemically heterogeneous forebrain structures often implicated in regulating social behaviors, stress, and feeding [44, 45]. Septal nuclei were recently demonstrated to receive sleep-related signals from the hippocampus in rats [46]. Chemogenetic activation of GABAergic neurons in the LS is sufficient to significantly increase NREM sleep amount [47]. The medial septum (MS) is repeatedly implicated in stress regulation that receives arousal-promoting projections from hypocretin-producing lateral hypothalamus neurons [48]. We report a significant increase in the number of cFos + cells of LS and the MS neurons following HRW treatment (Figure 7, Table 3). The directional relationship between observed sleep changes in HRW-treated mice and the *increase* in cellular activity in the forebrain structures reported here is unclear, though it is possible that these changes reflect the activity of local inhibitory interneurons.

Hypothalamic nuclei have long been thought to play diverse, important roles in sleep and arousal [49–51]. GABAergic and galanergic neurons in the preoptic area send inhibitory projections to important arousal-promoting nuclei, including the histaminergic neurons of the tuberomammillary nucleus (TMN) [49]. Optogenetic stimulation of these preoptic area neurons leads to increased SWA in mice. Chemogenetic activation of galanergic neurons in the VLPO significantly reduces sleep latency. Kroeger et al. [50] and others [for a review, 73] suggest that inhibitory neurons in the preoptic area may be central to the homeostatic organization of mammalian sleep. Here we report that HRW is sufficient to increase several important markers of sleep pressure but, importantly, we report no alterations to NREM SWA.

Inadequate sleep is diversely problematic for individuals and society. Chronic sleep loss shortens life and health span [8–10] and increases the risk of motor vehicle accidents [42]. Complex environmental, behavioral, and socioeconomic factors can make it difficult for people to routinely get sufficient opportunities for rest. Sleep disorders like insomnia make regular sleep difficult, even with sufficient opportunity. Emerging technologies offer personalized insights, and researchers access to ecologically valid sleep assessment [52], yet treatment options for poor and disordered sleep are limited, despite long-time public and scientific interest [13, 53].

Hydrogen is the most abundant chemical substance on the planet. As a therapeutic, hydrogen gas (H_2_) is nontoxic and has no known lethal dose or deleterious side effects. It has demonstrated the capacity to reduce oxidative stress, upregulate important immunological pathways, offset the effects of chemical and physiological challenges, and improve various metabolic conditions [39]. Significant sleep disturbances are common in patients with Parkinson’s disease (PD), with insomnia and daytime sleepiness frequently reported by patients and bed partners [54]. 6-hydroxydopamine (6-OHDA)-induced Parkinson’s disease is a frequently used rat model of PD, typified by the selective and rapid destruction of catecholaminergic neurons by administration of a neurotoxin [55]. Acute administration of HRW immediately pre- and post-administration of 6-OHDA is sufficient to (a) ameliorate the dopaminergic cell loss that typifies this model of PD and (b) alter dopamine-related behavior [20]. The substantia nigra, where dopamine is produced, and whose deterioration is a hallmark of PD, is an important sleep-regulatory region with essential roles in regulating arousal and REM sleep [56]. Together, acute administration of HRW has demonstrated the ability to regulate sleep-dependent processes (immunity and metabolism), and to act as a neuroprotectant in known sleep regulatory regions during a chemical challenge. These observations may underlie the results reported in this study.

## Conclusion

HRW has tractable effects on sleep–wake architecture and sleep consolidation. It decreases the amount of time for mice to fall asleep after light onset and it reduces sleep–wake fragmentation. During recovery from sleep deprivation, HRW increases both NREM and REM sleep amounts. HRW also increased neuronal activation in sleep regulatory regions of the brain, particularly in the VLPO and MnPO. Since neuronal activation in these brain areas is associated with promoting sleep, these results suggest that the effects of HRW are altering the neuronal regulation of sleep states, and may be a promising supplement for individuals experiencing from poor sleep efficiency.

## Limitations

This direct investigation of HRW and sleep in mice is the first of its kind. Every effort was taken to ensure the responsible assessment of HRW’s ability to influence arousal states and the recovery from sleep loss, including clearly defined positive controls for back testing, behavioral (sham) environmental disruptions to avoid unintended consequences of bottle replacements, blinded scoring of arousal behavior, and regular intra-scorer reliability testing to confirm very high agreement (>95%) among sleep scorers (see *Methods*). One limitation of this work is the ad libitum administration of HRW during the sleep assessment experiments. This route of administration was intentionally chosen as alternative administration routes that would provide greater control of dosage and timing (e.g. oral gavage) are anxiogenic and would significantly confound our assessment of arousal state by PSG. We believe our implementation of ad libitum administration reasonably reflects a possible real-world condition; though, admittedly, it is more likely that HRW would be administered in single doses at one or multiple times per day as has been seen in human trials [57–59].

Determining the relative dose of molecular hydrogen between mice and humans is complex, as several factors may be at play regarding molecular hydrogen’s therapeutic abilities. Significant evidence points to H_2_ working as a mitohormetic effector [14], as well as working in a dose-dependent manner [60]. As with all hormetic stressors, there will eventually be a plateau and decline when the stress is too significant, but this decline has not been observed in research thus far. The relative dosage of H_2_ could be calculated based on the average weight of the mice and average consumption of water and this could be compared to human consumption in clinical trials (in mg/H_2_). However, a one-to-one comparison of mg/H_2_ consumed may misrepresent physiologically relevant dosage, as relevant differences between humans and mice extend beyond mass. For example, H_2_ has been hypothesized to drive liver homeostasis [61]. Pharmacokinetic research has shown that a percentage of the H_2_ entering the liver is metabolized in an as yet unknown way. Metabolic rates are estimated to be 12.3× higher in mice than in humans [62] and it is currently unknown how or if liver metabolism changes the pharmacokinetic properties of H_2,_ or increases its therapeutic properties.

Oral gavage (and sham gavage) for the IHC study reported here enabled a greater level of temporal control over HRW dosing. However, as there are previous demonstrations of dose-dependent effects of HRW in various models [39], it is possible that the regions implicated by acute administration by gavage are wholly separate from the unknown mechanism(s) underlying changes to sleep behavior following ad libitum administration. The results of the experiments described above sufficiently justify the use of more invasive in vivo tools with cell-type and region specificity (e.g. optogenetics and chemogenetics), employed in parallel with PSG, to clarify the role of sleep regulatory systems in HRW-associated sleep changes. Finally, while immediate early gene activity reported here does not provide cell-type specific information, these results represent an important first step toward elucidating the mechanisms underlying the sleep-promoting effects of HRW in mice.

## Supplementary Material

zpad057_suppl_Supplementary_Figures_S1-S3_Tables_S1
