# Supplementary material for: Hydrogen-rich water improves sleep consolidation and enhances forebrain neuronal activation in mice
Source: Sleep Adv. 2023 Dec 30;5(1):zpad057. doi: 10.1093/sleepadvances/zpad057 (PMC10803172; doi:10.1093/sleepadvances/zpad057)
Supplement: zpad057_suppl_Supplementary_Figures_S1-S3_Tables_S1 [file zpad057_suppl_supplementary_figures_s1-s3_tables_s1.docx]

**Title:** Hydrogen-rich water improves sleep consolidation and enhances forebrain neuronal activation in mice

**Authors:** Vincent, S.^1^, Madani M.^1^, Dikeman D.^1^, Golden, K.^1^, Crocker, N.^1^, Jackson, C.^1^, Wimmer, S.^1^, Dover, M.^1^, Tucker, A. ^1^, Ghiani, C.A. ^2,3^, Colwell, C.S.^3^, LeBaron, T.W.^4,5^, Tarnava, A.^6^, Paul, K.^1*^

^1^ Department of Integrative Biology and Physiology, University of California Los Angeles, Los Angeles, CA, USA

^2^ Department of Pathology and Laboratory Medicine, David Geffen School of Medicine, University of California Los Angeles, Los Angeles, CA, USA

^3^ Department of Psychiatry and Biobehavioral Sciences, David Geffen School of Medicine, University of California, Los Angeles, Los Angeles, CA, USA

^4^ Department of Kinesiology and Outdoor Recreation, Southern Utah University, Cedar City, UT, USA

^5^ Molecular Hydrogen Institute, Enoch, UT, USA

^6^ Natural Wellness Now Health Products Inc, Maple ridge, BC, Canada

* Ketema Paul, 650 Charles E Young Dr. Rm 67-362, Los Angeles, CA 90095, ketema.paul@ucla.edu

**Figure S1. Visualization of experimental protocol for sleep experiments.**


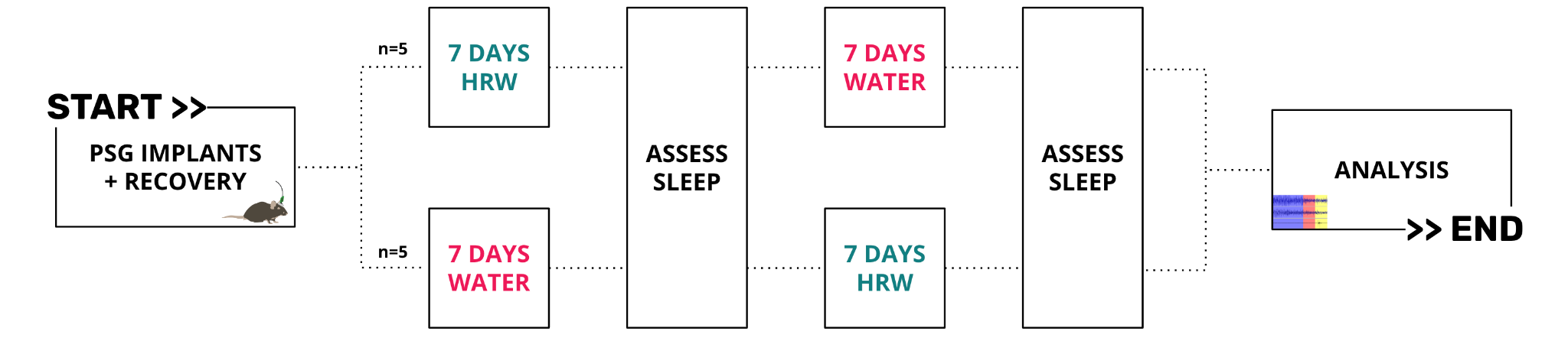


**Figure S2. Visualization of experimental protocol for IHC experiments.**

**
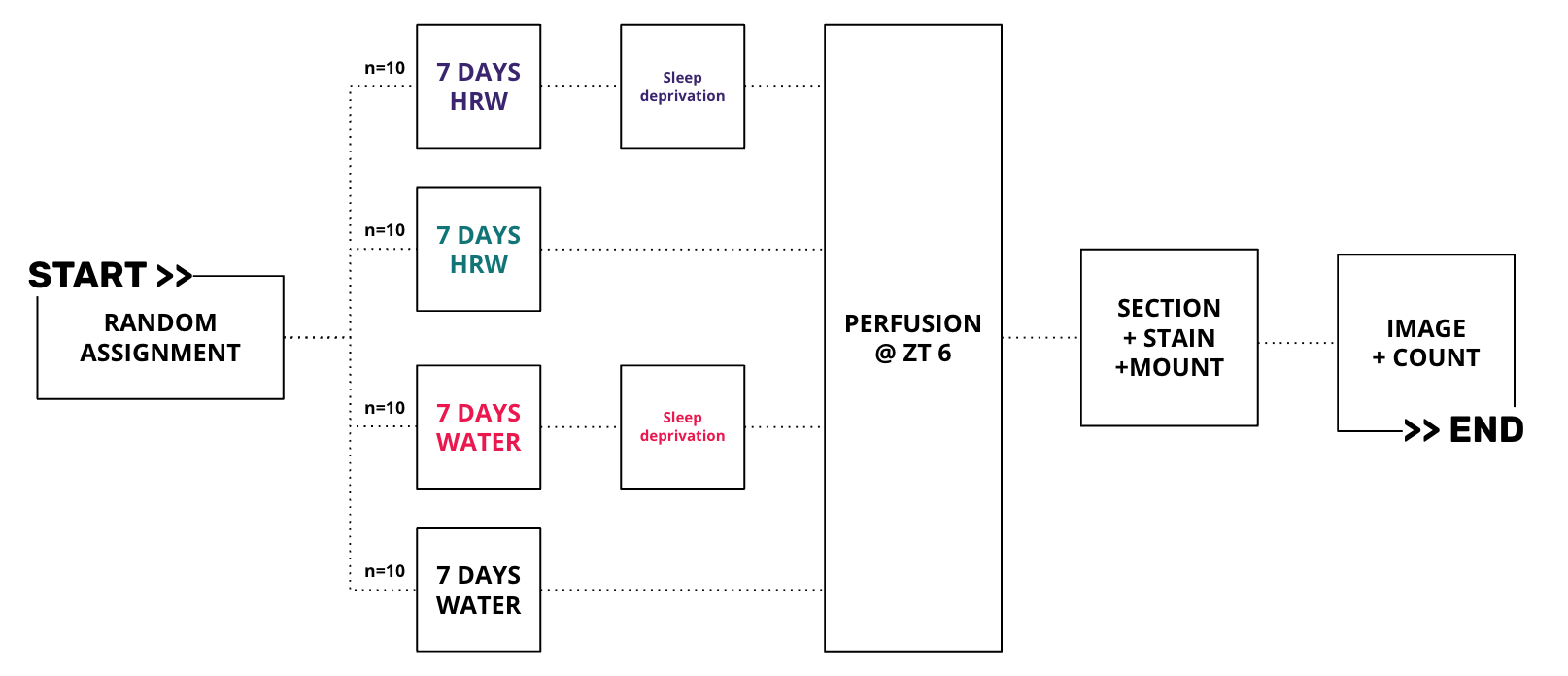
**

**Figure S3. Expected rebound of NREM relative delta power is observed in EEG2 in untreated and HRW-treated conditions.**


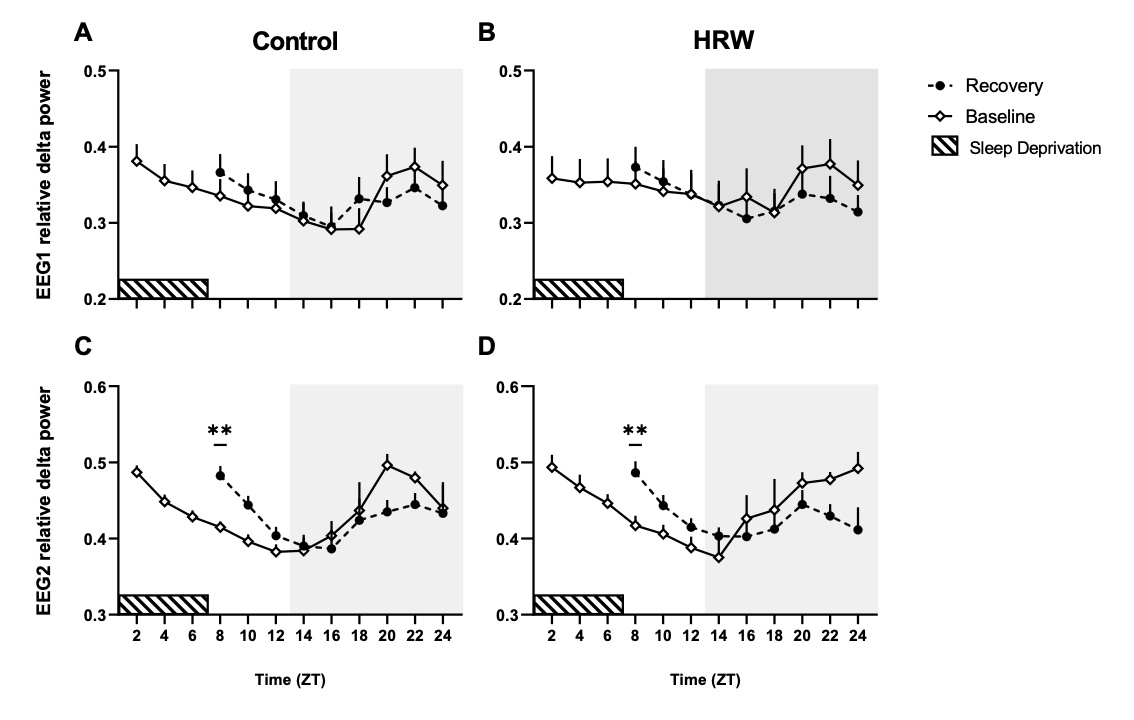


**Table S1. Full three-way ANOVA statistics for brain regions of interest.**

| **Source of Variation** | **SS** | **F(DFn, DFd)** | **P value** | **Summary** |
| --- | --- | --- | --- | --- |
| ***Diagonal Band (DB)***  Sex  Treatment  Sleep  Sex x Treatment  Sex x Sleep  Treatment x Sleep  Sex x Treatment x Sleep | 162.7  1175  1032  0.0745  421.1  800  80.53 | *F* (1, 53) = 0.4967  *F* (1, 53) = 3.5900  *F* (1, 53) = 3.1530  *F* (1, 53) = 0.0002  *F* (1, 53) = 1.2860  *F* (1, 53) = 2.4430  *F* (1, 53) = 0.2459 | *P* = 0.4840  *P* = 0.0636  *P* = 0.0815  *P* = 0.9880  *P* = 0.2619  *P* = 0.1240  *P* = 0.6220 | ns  ns  ns  ns  ns  ns  ns |
| ***Medial Septum (MS)***  Sex  Treatment  Sleep  Sex x Treatment  Sex x Sleep  Treatment x Sleep  Sex x Treatment x Sleep | 147.1  1029  2131  448.7  239.5  697.1  67.04 | *F* (1, 65) = 1.4390  *F* (1, 65) = 10.060  *F* (1, 65) = 20.840  *F* (1, 65) = 4.3880  *F* (1, 65) = 2.3420  *F* (1, 65) = 6.8160  *F* (1, 65) = 0.6556 | *P* = 0.2347  *P* = 0.0023  *P* < 0.0001  *P* = 0.0401  *P* = 0.1308  *P* = 0.0112  *P* = 0.4211 | ns  **  ****  *  ns  *  ns |
| ***Lateral Septum (LS)***  Sex  Treatment  Sleep  Sex x Treatment  Sex x Sleep  Treatment x Sleep  Sex x Treatment x Sleep | 24594  202804  155038  3052  7031  142362  45818 | *F* (1, 63) = 2.8320  *F* (1, 63) = 23.350  *F* (1, 63) = 17.850  *F* (1, 63) = 0.3514  *F* (1, 63) = 0.8096  *F* (1, 63) = 16.390  *F* (1, 63) = 5.2760 | *P* = 0.0974  *P* < 0.0001  *P* < 0.0001  *P* = 0.5554  *P* = 0.3717  *P* = 0.0001  *P* = 0.0250 | ns  ****  ****  ns  ns  ****  * |
| ***Ventrolateral Preoptic Nucleus (VLPO)***  Sex  Treatment  Sleep  Sex x Treatment  Sex x Sleep  Treatment x Sleep  Sex x Treatment x Sleep | 10.50  75.67  163.10  39.90  0.6156  101.5  1.180 | *F* (1, 64) = 0.7804  *F* (1, 64) = 5.6230  *F* (1, 64) = 12.120  *F* (1, 64) = 2.9650  *F* (1, 64) = 0.0457  *F* (1, 64) = 7.5440  *F* (1, 64) = 0.0877 | *P* = 0.3803  *P* = 0.0207  *P* = 0.0009  *P* = 0.0899  *P* = 0.8313  *P* = 0.0078  *P* = 0.7681 | ns  *  ***  ns  ns  *  ns |
| ***Median Preoptic Area (MnPO)***  Sex  Treatment  Sleep  Sex x Treatment  Sex x Sleep  Treatment x Sleep  Sex x Treatment x Sleep | 7.102  537.4  406.4  189.0  4.002  745.0  1.613 | *F* (1, 62) = 0.1920  *F* (1, 62) = 14.530  *F* (1, 62) = 10.990  *F* (1, 62) = 5.1110  *F* (1, 62) = 0.1082  *F* (1, 62) = 20.150  *F* (1, 62) = 0.0436 | *P* = 0.6627  *P* = 0.0003  *P* = 0.0015  *P* = 0.0273  *P* = 0.7433  *P* < 0.0001  *P* = 0.8353 | ns  ***  **  *  ns  ****  ns |

**Supplementary Figure Captions:**

**Figure S1. Visualization of experimental protocol for sleep assessment.**

**Caption:** Visualization of the experimental protocol for assessing the effects of HRW on sleep.

**Figure S2. Visualization of experimental protocol for IHC experiments.**

**Caption:** Visualization of the experimental protocol for assessing the effects of HRW on neuronal activation in the brain using IHC.

**Figure S3.  Expected rebound of NREM relative delta power following sleep deprivation is observed in EEG2 in untreated and HRW-treated conditions.**

**Caption:** Multiple paired t-test following 6 hours of acute sleep deprivation, mice experience an expected increase in slow wave activity (relative delta power) in EEG2 in both the untreated condition (*P*=0.00057, *t*=5.189, *df*=9) and following 6 hours of sleep deprivation (*P*=0.00039, *t*=5.834, *df*=8**)**. Icons represent the mean and error bars are standard error of the mean. Shaded boxes label the dark phase. **, *P*<0.01.

**Supplementary Table Caption:**

**Table S1: Full three-way ANOVA statistics for brain regions of interest.**

**Caption:** Three-way ANOVA with sex, treatment, and total sleep as factors in brain regions of interest. No main effect of sleep was revealed. A Bonferroni post-test confirmed.
